# Prehospital Surgical Airway Management Skills in a Rural Emergency Medical Service System

**DOI:** 10.7759/cureus.41864

**Published:** 2023-07-14

**Authors:** Brian L Risavi, Jestin Carlson, Erin M Reese, Aaron Raleigh, Jordan Wallis

**Affiliations:** 1 Emergency Medicine, Lake Erie College of Osteopathic Medicine, Erie, USA; 2 Emergency Medicine, Allegheny Health Network, Erie, USA; 3 Emergency Medicine, UPMC (University of Pittsburgh Medical Center) Hamot, Erie, USA

**Keywords:** airway management, cricothyrotomy, ems, prehospital, surgical airway

## Abstract

Background: The objective of this study is to describe the education, training, and use of prehospital surgical airways in a rural Emergency Medical Service (EMS) system.

Materials and methods: We conducted an internet-based survey instrument of all advanced life support (ALS) EMS agencies in a seven-county rural EMS system in Pennsylvania. ALS agencies were queried regarding basic demographic information as well as the number of surgical airways performed in the previous 10 years as well as the education and training of EMS providers in surgical airways.

Results: The survey was completed by 11 of 20 ALS EMS agencies in our region (55% rate of return). The content and frequency of training varied considerably among EMS agencies. Only four prehospital surgical airways were performed during the study period. One patient survived to hospital discharge to home.

Conclusion: Surgical airways are an infrequently performed procedure in the rural prehospital setting. There is no universally accepted standard for teaching or evaluating the competency of this potentially life-saving procedure. Further efforts to establish a core educational curriculum appear warranted.

## Introduction

Surgical airways are infrequently performed in the rural prehospital setting, both in adult and pediatric patients [1-4]. A study of 16 states revealed that only 88 prehospital surgical airways were performed in 2,333,254 patients [4]. In Pennsylvania, paramedics must be authorized by their medical director to perform surgical airways [5]. Moreover, there is no curriculum for testing surgical airway techniques at the National Registry of Emergency Medical Technicians (NREMT) level [6]. Additionally, there is no uniform training standard for teaching this important skill. Given the low frequency of prehospital surgical airways, skill decay is an ongoing concern [7].

Training poses an additional consideration for organizations when deciding what device or technique to implement, as more complex techniques lend to more rapid attrition of procedural skills [8,9]. However, given the infrequent use and critical implications when deployed, frequent high-quality training is necessary regardless of the technique selected. In educating Emergency Medical Service (EMS) providers in surgical airways, the goal is to achieve mastery learning in this complex procedure with a significant learning curve [10]. Deliberate practice is utilized to achieve specific goals, provide immediate feedback, and provide sufficient time to practice the skill [11,12]. Mastery learning utilizes deliberate practice plus an assessment of specific performance criteria in accordance with established minimum standards to achieve an expert level of performance [13].

While these practices may be utilized in high-resource settings, rural programs may have even less clinical exposure to surgical airways and limited resources for training. How often these skills are utilized in the clinical setting and how rural agencies train on surgical airways is unknown. The purpose of this study is to describe the education, training, and use of prehospital surgical airways in a rural EMS system.

## Materials and methods

Local Institutional Review Board approval was obtained from the Lake Erie College of Osteopathic Medicine (Protocol #28-128) for this study. An internet-based survey of all advanced life support (ALS) EMS agencies in a seven-county rural EMS system in Pennsylvania was conducted, utilizing one survey per EMS agency (see Appendix). The population of the seven-county EMS region is 598,721 (average: 85,531 per county; range: 6,973-270,876). The authors sought to ascertain specific information regarding the education, training, and use of surgical airways in a rural EMS system. All ALS agencies in our region were queried regarding basic demographic data, such as call volume, number of ALS providers, and number of surgical airways performed in the previous 10 years. Additionally, the frequency and nature of surgical airway training as well as the person(s) conducting the training were collected. The types of surgical airway devices, including the use of the bougie, were evaluated also. Finally, the number of actual surgical airways performed, including patient outcome, was collected. Each EMS agency was permitted to complete the survey once with an IP address used for verification. The survey was not distributed to any other organizations or individuals. Participation in the survey was voluntary and no incentives were offered for completion of the survey.

## Results

The survey was completed by 11 of 20 ALS services in the region (55% rate of return). The number of paramedics functioning in EMS agencies in the region ranged from three to 100 (average 18.4). EMS agency call volume varied. See Table 1 for a data summary. There were 54.55% of EMS agencies with call volumes less than 5,000 patients/year. There were 27.27% of EMS agencies with 5,000-10,000 patients/year. No EMS agencies with patient volumes between 10,001 and 15,000 were noted. Finally, there were 18.18% of EMS agencies with call volumes greater than 15,000. The training was conducted by the EMS agency medical director in six (55%) of the EMS agencies and by other staff appointed by the medical director, such as training officers, in the remaining EMS agencies. The training was largely conducted by lecture and hands-on simulation using manikins or pig tracheas. The educational content was developed by the respective EMS agencies as there is no standardized curriculum for prehospital surgical airways. No videos or podcasts were used. Duration of the training ranged from less than 1 hour to greater than 2 hours, with the majority between 1 and 2 hours (72.7%). The frequency of training was variable. Seven EMS agencies (63%) conducted training annually, and twice yearly in one (9%). One EMS agency conducted training twice in the previous five years and one EMS agency conducted training once in four years. The last EMS agency indicated that training had been done annually. With respect to the time interval since the last surgical airway training was completed: There were two EMS agencies that did not conduct training for greater than three years. Five EMS agencies conducted training in the previous one to three years, while four EMS agencies conducted training within the past year. Seven EMS agencies (63%) used a bougie for surgical airways. Nearly 10% of services were not permitted to perform surgical airways. Only four surgical airways were performed in the prehospital setting in the region in the past 10 years. All were adult trauma patients, with only one patient surviving to discharge (to home).

**Table 1:** Summary of Data

Survey Questions		Total (%)	Total (n)
Annual EMS Agency Call Volume	Less than 5,000	54.5	6
	5,000-10,000	27.27	3
	10,001-15,000	0	0
	Greater than 15,000	18.18	2
EMS Agency Authorization by Medical Director	Yes	90.91	10
	No	9.09	1
Duration of Training Session	Less than 60 minutes	18.18	2
	1-2 hours	72.73	8
	Greater than 2 hours	9.09	1
Frequency of Training	Annually	63.64	7
	Semi-annually	9.09	1
	Other	27.27	3
Time Interval Since Last Surgical Airway Training	Less than 1 year	36.36	4
	Between 1-3 years	45.45	5
	Greater than 3 years	18.18	2

## Discussion

Prehospital surgical airway skills are rarely performed but remain important in the skill set of ALS providers with respect to advanced airway management [3]. In fact, many EMS personnel feel inadequately trained in the procedure [14]. Military data indicate that airway-related issues remain the third leading cause of preventable death on the battlefield [7]. In the civilian sector, many patients requiring surgical airways do not survive [15-18]. Generally speaking, the most difficult aspect of performing a surgical airway is recognizing the need to do it [19]. The stress associated with a high-impact, low-frequency procedure can be significant, and may have an impact on clinical decision-making [20,21].

Equally important are non-technical skills such as teamwork and time management. Crew resource management is important in performing the procedure rapidly and efficiently [22]. Crew resource management, as the name implies, relates to the human (non-technical) factors associated with teamwork in high-stress/time-critical situations that contribute to error, resulting in patient harm [23,24]. Training in crew resource management may improve leadership skills, problem-solving, communication, situational awareness, and teamwork [25]. Simulation plays a significant role in advancing these parameters, particularly in the younger generation of learners [26]. When the need for a surgical airway arises, one must be able to recognize the need and respond quickly and efficiently. Communication is key in utilizing resources to secure the airway rapidly [27]. Situational awareness is important in anticipating the equipment needs for securing the airway via surgical means [24]. Knowing where the equipment is located, having the ventilatory equipment at the ready, and anticipating complications are all important considerations.

The bougie-assisted surgical airway was described in 2010 and appears to be faster than conventional teaching and demonstrated fewer complications [28,29]. Conventional teaching often fails when the airway tube is inserted into the subcutaneous tissue [30]. Bougie use was found to be more effective than a standard rigid stylet [31]. The bougie appears to be helpful in performing a surgical airway using a conventional endotracheal tube due to the curvature of the tube [31,32].

Teaching surgical airway skills is important; however, many devices and methods exist. Some of the well-known kits include Melker™, QuickTrach™, Minitrach II, and the Portex® Cricothyrotomy Kit (PCK) [33]. These commercial kits have been compared to various open standard surgical techniques, as well as among themselves, with mixed results in efficacy, speed, complications, and operator preference [8,9,34]. Thus, there is a need for formal surgical airway training in EMS to ensure competency in airway crisis situations.

Limitations

The results represent one rural EMS system in one state. Results may not be generalizable to all EMS systems. One EMS agency representative completing the survey for the organization is also a potential limitation. Our sample size included only four surgical airways done in the past 10 years in our region. The retrospective nature of this survey instrument introduces the potential for bias.

## Conclusions

Surgical airways are an infrequently performed procedure in the rural prehospital setting. Education and training vary considerably, both in content and frequency. A significant number of EMS providers are not using the bougie as part of surgical airway training despite literature supporting its use. There is no universally accepted standard for teaching or evaluating the competency of this potentially life-saving procedure. Further efforts to establish a core educational curriculum and mechanism for evaluating provider competency appear warranted.

## Appendices

Survey questions

What is the annual call volume (calls per year) of your EMS agency? (Circle one)

            Less than 5,000            5,000-10,000             10,000-15,000            Greater than 15,000

Is your EMS agency authorized by your EMS agency medical director to perform surgical airways in the prehospital setting? This does not include transtracheal jet ventilation with a needle.

            Yes                  No       (Circle one)

Is your EMS agency authorized by your EMS agency medical director to perform needle cricothyrotomy with a jet ventilator?

            Yes                  No       (Circle one)

How many paramedics (full or part-time) are currently functioning in your EMS agency? _________          

Who conducts the training for surgical airways for your prehospital providers?

            EMS agency  medical director            EMS agency chief/director       Other___________

How is the training conducted? (circle all that apply)

            Lecture

            Hands-on with simulation equipment (pig tracheas, manikin, etc)

            Video/podcast

Who developed the training content?

            EMS agency medical director             EMS agency chief/director        Other____________

What is the duration of the training session? (circle one)

            Less than 60 minutes                           1-2 hours                                 Greater than 2 hours

How frequently is the training conducted? (Circle one)

            Annually                     Semi-annually             Other__________________

How long has it been since your EMS providers participated in surgical airway training? (Circle one)

            Less than one year      Between 1-3 years       Greater than 3 years      

What equipment is used for surgical airways?

            Commercially available kit

            Scalpel and endotracheal tube, Shiley trach tube, etc.

            Other______________________

Do your EMS providers use a bougie for surgical airways? (Circle one)

            Yes                  No

How many times has your EMS agency performed an actual surgical airway in the past 10 years?______          

            For each surgical airway complete the following questions:

Surgical Airway #1

              Was this a trauma or medical patient? (Circle one)

              Was surgical airway placement successful?

                        Yes                  No

              Was the patient in cardiac arrest prior to surgical airway attempt?

                        Yes                  No           

               Did the patient survive?

                        Yes                  No

                Was the patient’s neurologic status impaired compared to prior baseline?

                        Yes                   No

Surgical Airway #2

                 Was this a trauma or medical patient? (Circle one)

                 Was surgical airway placement successful?

                        Yes                  No

                 Was the patient in cardiac arrest prior to surgical airway attempt?

                        Yes                  No

                  Did the patient survive?

                        Yes                  No

                   Was the patient’s neurologic status impaired compared to prior baseline?

                        Yes                   No

 Surgical Airway #3

                    Was this a trauma or medical patient? (Circle one)

                    Was surgical airway placement successful?

                        Yes                  No

                    Was the patient in cardiac arrest prior to surgical airway attempt?

                        Yes                  No

                    Did the patient survive?

                        Yes                  No

                     Was the patient’s neurologic status impaired compared to prior baseline?

                        Yes                   No

Surgical Airway #4

                      Was this a trauma or medical patient? (Circle one)

                      Was surgical airway placement successful?

                         Yes                  No

                       Was the patient in cardiac arrest prior to surgical airway attempt?

                          Yes                  No

                        Did the patient survive?

                          Yes                  No

                        Was the patient’s neurologic status impaired compared to prior baseline?

                           Yes                   No

Surgical Airway #5

                         Was this a trauma or medical patient? (Circle one)

                         Was surgical airway placement successful?

                             Yes                  No

                          Was the patient in cardiac arrest prior to surgical airway attempt?

                              Yes                  No

                           Did the patient survive?

                              Yes                  No

                            Was the patient’s neurologic status impaired compared to prior baseline?

                               Yes                   No

Surgical Airway #6

                             Was this a trauma or medical patient? (Circle one)

                             Was surgical airway placement successful?

                                 Yes                  No

                             Was the patient in cardiac arrest prior to surgical airway attempt?

                                Yes                  No

                              Did the patient survive?

                                 Yes                  No

                              Was the patient’s neurologic status impaired compared to prior baseline?

                                  Yes                   No

Surgical Airway #7

                               Was this a trauma or medical patient? (Circle one)

                               Was surgical airway placement successful?

                                   Yes                  No

                               Was the patient in cardiac arrest prior to surgical airway attempt?

                                   Yes                  No

                                Did the patient survive?

                                   Yes                  No

                                 Was the patient’s neurologic status impaired compared to prior baseline?

                                     Yes                   No

Surgical Airway #8

                                 Was this a trauma or medical patient? (Circle one)

                                 Was surgical airway placement successful?

                                     Yes                  No

                                 Was the patient in cardiac arrest prior to surgical airway attempt?

                                      Yes                  No

                                  Did the patient survive?

                                     Yes                  No

                                  Was the patient’s neurologic status impaired compared to prior baseline?

                                      Yes                   No

Surgical Airway #9

                                  Was this a trauma or medical patient? (Circle one)

                                  Was surgical airway placement successful?

                                      Yes                  No

                                   Was the patient in cardiac arrest prior to surgical airway attempt?

                                       Yes                  No

                                     Did the patient survive?

                                        Yes                  No

                                      Was the patient’s neurologic status impaired compared to prior baseline?

                                         Yes                   No

Surgical Airway #10

                                       Was this a trauma or medical patient? (Circle one)

                                       Was surgical airway placement successful?

                                           Yes                  No

                                       Was the patient in cardiac arrest prior to surgical airway attempt?

                                           Yes                  No

                                        Did the patient survive?

                                           Yes                  No

                                        Was the patient’s neurologic status impaired compared to prior baseline?

                                            Yes                   No

Thank you for your participation!                      
